# Prehospital FAST reduces time to admission and operative treatment: a prospective, randomized, multicenter trial

**DOI:** 10.1007/s00068-021-01806-w

**Published:** 2021-10-18

**Authors:** Benjamin Lucas, Dorothea Hempel, Ronny Otto, Franziska Brenner, Mario Stier, Ingo Marzi, Raoul Breitkreutz, Felix Walcher

**Affiliations:** 1 Department of Trauma Surgery, Otto-Von-Guericke University Magdeburg, Leipziger Str. 44, 39120 Magdeburg, Germany; 2 Central Emergency with Admission Ward, Otto-Von-Guericke University Magdeburg, Magdeburg, Germany; 3 Clinic of Nephrology and Hypertension, Diabetes and Endocrinology, Otto-Von-Guericke University Magdeburg, Magdeburg, Germany; 4 Emergency Department, Asklepios Klinik Wandsbek, Alphonsstr. 14, 22043 Hamburg, Germany; 5 Department of Trauma Surgery, Johann Wolfgang Goethe-University, Frankfurt, Germany; 6 Department of Hand Surgery, Agaplesion Diakonieklinikum Hamburg Gemeinnützige GmbH, Hamburg, Germany; 7 Institute for Health and Social (IfGS), FOM University of Economy and Management, Frankfurt Campus, Frankfurt, Germany

**Keywords:** Abdominal injury, FAST, Prehospital ultrasound, Time-to-surgery, Trauma room

## Abstract

**Background:**

The focused assessment with sonography in trauma (FAST) exam is an established trauma care diagnostic procedure. Ultrasound performed during prehospital care can improve early treatment and management of the patients. In this prospective randomized clinical trial, we wanted to assess whether a pre-hospital FAST (p-FAST) influences pre-hospital strategy and the time to operative treatment.

**Methods:**

We studied 296 trauma victims in a prehospital setting. Inclusion criteria were potential abdominal injuries identified either by clinical examination or suggested by the mechanism of injury. Physician-staffed helicopters and emergency ambulances were equipped with portable ultrasound devices. According to a scheme related to calendar weeks, a clinical exam only (CEX) or a clinical exam together with a p-FAST (CEX-p-FAST) was conducted. Outcome variables were prehospital diagnosis and strategy, the time to admission to the trauma room and to operation theater. The study was approved by the university ethical committee (REB#: 46/06).

**Results:**

CEX-p-FAST showed a high sensitivity (94.7%) and specificity (97.6%) in detection of free fluid compared to CEX-only (80.0%, 84.4%). The median time to admission was reduced significantly by 13 min and to operative treatment by 15 min after CEX-p-FAST. We observed a cross-over rate of 30.8% of p-FAST (*n* = 36) to CEX-p-FAST during the CEX-only weeks.

**Conclusion:**

According to the experience of the principal investigators, CEX-p-FAST was superior to CEX-only. Despite the time needed for p-FAST, the relevant admission time was significantly shorter. Thus, p-FAST is recommended in addition to CEX if possible for decision-making in prehospital trauma care.

**Trial registration:**

German Clinical Trials Register #DRKS00022117—Registered 10 July 2020—Retrospectively registered, https://www.drks.de/drks_web/navigate.do?navigationId=trial.HTML&TRIAL_ID=DRKS00022117.

## Background

Ultrasound is an integral part of the diagnostic algorithm after blunt trauma and a class I recommendation for in-hospital assessment as it can reduce the time to emergency surgery [1]. The focused assessment with sonography in trauma (FAST) exam is an adjunct of the “Advanced Trauma Life Support” (ATLS) algorithm [2, 3]. Due to the miniaturization of devices, prehospital ultrasound, such as prehospital FAST (p-FAST), has been available for over a decade [4]. Despite the technical possibilities, the use of prehospital ultrasound is not comprehensively applied [5, 6]. Factors limiting the use of prehospital ultrasound are the cost linked with the acquisition of the devices, the training of the emergency medical services (EMS) staff, and a lack of scientific evidence that morbidity and mortality can be reduced significantly. Test characteristics have been shown to be between 90 and95% for sensitivity and 90 and100% for specificity for the detection of free abdominal fluid [7]. It has also been shown that independent of the underlying subspecialty, a brief training program is sufficient to acquire the necessary skills to use a prehospital ultrasound [8, 9].

Several studies have suggested that p-FAST can reduce the time to operative treatment, improve the understanding of the severity of injury resulting in changes in the preparation and the management of EMS at the receiving hospital [4, 10, 11]. In a study on 71 patients, Bodnar et al. found that a positive p-FAST leads to a significantly shorter time to definite treatment than no p-FAST or negative p-FAST [10]. The main criticism of using p-FAST regarding its direct impact on patient outcomes remains unresolved [12].

Thus, in this prospective randomized trial, our aim was to assess the impact of p-FAST on prehospital diagnostic and strategy as well as the time to operative treatment.

## Patients, materials and methods

### Study design and aim

We designed a prospective, randomized, multicenter trial. The aim of the study was to analyze the influence of p-FAST on the time to admission to the trauma room and time to surgery, if necessary. This was measured as the time from the examination at the scene to admission to the trauma room or operation theater.

**Fig.1:**
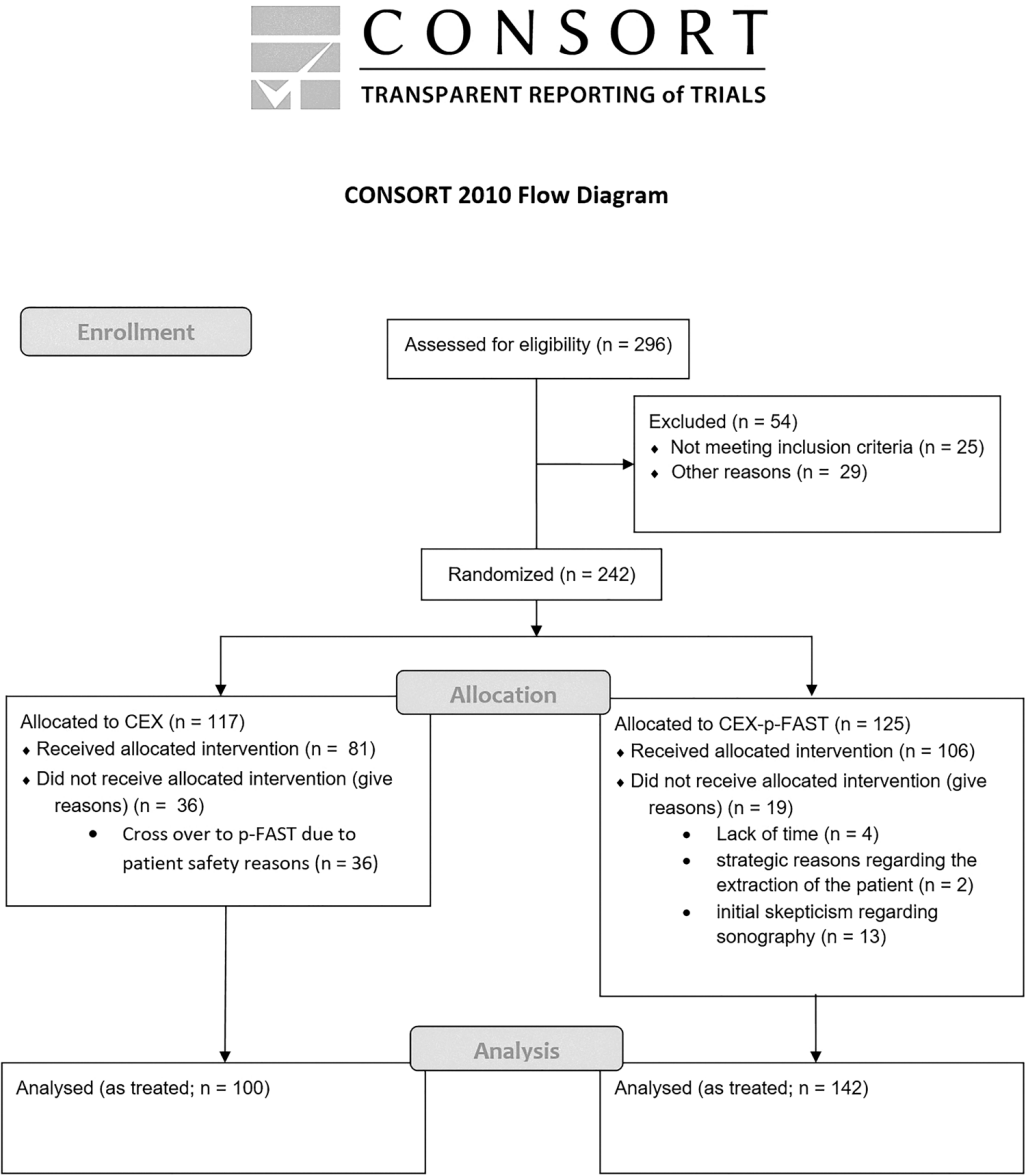
Consort flow diagram. CONSORT flow diagram of enrollment, allocation, and analysis

Our secondary aim was to evaluate the effect of the p-FAST on the prehospital treatment strategy. Therefore, we analyzed the level of care (level of trauma center) that was chosen after p-FAST, the information given to the receiving hospital, and the prehospital treatment strategy. The methodology used in this study adhered to the CONSORT statement [13] (Fig. 1). The study was approved by the ethics committee of the University Hospital of Frankfurt, Frankfurt am Main, Germany (REB#: 46/06).

The study was registered retrospectively in the German Clinical Trials Register (#DRKS00022117).

### Prehospital emergency services

For this study, six emergency ambulances, staffed with an emergency physician (EP), and paramedic and one rescue helicopter were monitored (Table 2). The rescue teams were equipped with a hand-held ultrasound device (Primedic Handyscan, Metrax Company, Rottweil, Germany). All emergency ambulances had 24-h emergency physician coverage.

All EPs were instructed on the aims of the study by a research assistant who was available on the phone 24/7.

### Patient recruitment and randomization

Between April 2007 and December 2009, all trauma patients in whom a blunt abdominal trauma could not be excluded on the basis of clinical information or trauma mechanism were recruited for the study. There were no formal exclusion criteria in the study protocol. During even calendar weeks, the physicians were instructed to perform a p-FAST (CEX-p-FAST) in addition to their regular clinical exam (CEX). During odd calendar weeks, patients had to be assessed by standard CEX. This randomization is characterized as quasi-randomized.

### Data acquisition and outcome parameters

All data pertaining to CEX and CEX with additional P-FAST (CEX-p-FAST) were documented in a case report form (CRF). The CRF included the findings of the CEX and the CEX-p-FAST assessment, as well as changes in the treatment strategy, the information given to the receiving hospital and the level of care provided by the receiving hospital. Computer tomography of the abdomen (CT-abdomen) was performed in all patients as the gold standard for diagnosing free fluid and injury to parenchymal organs. During the stay in the trauma room, the CRFs were completed by an assistant who documented the exact times of all interventions and operative procedures. For primary outcome, the time to admission from the prehospital scenario (first examination in the prehospital scenarios to arrival in trauma room) and the time to operative treatment (first examination in the prehospital scenarios to arrival in the operation theater) were analyzed. As secondary outcome, changes in the prehospital treatment strategy were examined. For changes in therapy, “permissive hypotension”, “small volume resuscitation”, “no volume replacement” and “establishing of further venous accesses” was documented. For changes in communication, “suspected free intraabdominal fluid”, “suspected abdominal trauma”, “ultrasound conducted”, “free intraabdominal fluid detected”, “ultrasound conducted, free intraabdominal fluid not detected” and “abdominal trauma not suspected” were documented.

### Statistical analysis

Sample size calculation was performed assuming a time benefit of 15 min with a standard deviation of 30 min. Assuming that about 20% of abdominal trauma patients present with free fluid in the abdomen, a sample size of 1000 patients was calculated for a power of 90% and a type II error of 10%. An interim analysis was planned after the inclusion of 500 patients.

Data were analyzed using the Statistical package for the social science, version 17.0 (SPSS 17.0, IBM, Armonk, USA). All data were presented as the mean ± standard deviation (SD) for normally distributed variables and as the median in case of non-normal distribution. For the comparison of two groups, we used Mann–Whitney *U *test. The normal distribution was tested by Kolmogorov–Smirnov tests. For categorical data, Fisher’s tests were used. *p *values lower than 0.05 were considered statistically significant.

## Results

At the end of 2009, we conducted an interim analysis. A cross-over rate of 30.8% from the CEX group to the CEX-p-FAST group was observed. Meaning that p-FAST was just used by the prehospital team even it should not have been used according to the study protocol. Analyzing these results, we observed an overwhelming acceptance of the p-FAST. However, physicians used the ultrasound even in the CEX group due to patient safety reasons. Therefore, the study had to be interrupted, since the protocol could not be performed as planned. After careful deliberations, the principal investigators decided that patients should not be deprived of receiving the best available tool in diagnostic after blunt trauma, and the study be discontinued due to ethical reasons. Therefore, the explorative data analysis includes all patients assessed until this time point and an as-treated analysis was performed.

### Patient characteristics

A total of 296 patients were included for analysis (Fig. 1). Fifty-four patients had to be excluded from the final analysis because of incomplete documentation (29), transfer to a non-participating hospital (9), ambulatory-only treatment (9), treatment solely at the scene (5) or death at the scene (2).

The remaining 242 patients consisted of 170 male (70.2%, mean age = 40.1 ± 19.4) and 64 female (26.4%, mean age = 41.3 ± 21.6) patients. The sex of 8 patients was not documented. The mean age of the study population was 40.5 ± 20.0 years (Table 1). Most patients were transported by the helicopter service of Christoph 2 (52.1%) and the emergency ambulance 4 (27.3%; Table 2).

**Table 1:** Patient characteristics

	Female (*n* = 64)	Male (*n* = 170)
Age (years)	41.3 ± 21.6	40.1 ± 19.4
CEX (*n*)	23	70
CEX-p-FAST (*n*)	41	100
CEX (years)	44.0 ± 24.0	41.2 ± 20.5
CEX-p-FAST (years)	39.8 ± 20.3	39.4 ± 18.8

**Table 2:** Participating emergency transport vehicles with respective hosting hospitals and the number of included patients

Emergency transport vehicle	Hosting hospital (level of trauma center)	Included patient absolute (relative)
NEF 1	BG Hospital Frankfurt (level 1)	24 (9.9%)
NEF 2	Nord-West Hospital Frankfurt (level 2)	15 (6.2%)
NEF 3	Hospital Frankfurt Höchst (level 1)	11 (4.5%)
NEF 4	University Hospital Frankfurt (level 1)	66 (27.3%)
RTH Christoph 2	BG Hospital Frankfurt (level 1)	126 (52.1%)

CEX-only was performed on 100 patients (41.3%) and CEX-p-FAST on 142 patients (58.7%). In six patients randomized to the CEX-p-FAST group, the p-FAST exam was not carried out. These findings were investigated by the respective EPs who documented that p-FAST could not be performed in 4 cases due to a lack of time and in 2 cases due to strategic reasons regarding the extraction of the patient.

The median injury severity score (ISS) was 14 in the CEX group and 17 in the CEX-p-FAST group (Kolmogorov–Smirnov *p* < 0.001; Mann–Whitney *U *test *p* = 0.883).

As receiving hospitals, only the level I trauma centers were chosen by the EPs. Eighty-seven (36.0%) of patients, *n* = 36 (36.0%) in the CEX group, and *n* = 51 (35.9%) in the CEX-p-FAST group arrived in the trauma room with the airway secured by an endotracheal tube. On admission to the trauma room, the treating physician of the receiving hospital classified *n* = 226 (93.4%) patients as stable and *n* = 16 (6.6%) as unstable.

### Free intraabdominal fluid

After the CEX or CEX-p-FAST examinations in the prehospital scenarios, free fluid was suspected in *n* = 22 patients in the CEX group (22.0%) and *n* = 21 patients in the CEX-p-FAST group (14.8%). Based on the results of the CT abdomen, *n* = 10 patients (10.0%) in the CEX group and *n* = 19 in the CEX-p-FAST group (13.4%) were diagnosed with a positive finding of free intraabdominal fluid. In the CEX group, there were *n* = 8 true positive, *n* = 14 false positive, and *n* = 2 false negative results. In the CEX-p-FAST group, there were *n* = 18 true positive, *n* = 3 false positive, and *n* = 1 false negative results (Table 3). The sensitivity for the CEX-p-FAST was higher (94.7%) than for the CEX group (80.0%). Table 4 shows detailed test characteristics of the sensitivity and specificity of CEX and CEX-p-FAST group controlled by CT.

**Table 3:** Findings in CEX and CEX-p-FAST in comparison to the computer tomography results

	CT positive	CT negative	
*CEX-p-FAST positive*	18	3	21
*CEX positive*	8	14	22
*CEX-p-FAST negative*	1	120	121
*CEX negative*	2	76	78
*CEX-p-FAST*	19	123	142
*CEX*	10	90	100

**Table 4:** Test statistics of the CEX and CEX-p-FAST assuming the computer tomography as the gold standard

	CEX (%)	CEX-p-FAST (%)
Sensitivity	80.0	94.7
Specificity	84.4	97.6
Positive predictive value	36.4	85.7
Negative predictive value	97.4	99.2

### Time to admission to the trauma room

An important measurement was the time to admission from the prehospital scenario. For all patients, the median transfer time from examination to admission to the trauma room was 33 min in the CEX group and 30 min in the CEX-p-FAST group (not significant, Kolmogorov–Smirnov *p* < 0.001; Mann–Whitney *U *test *p* = 0.365).

**Fig. 2:**
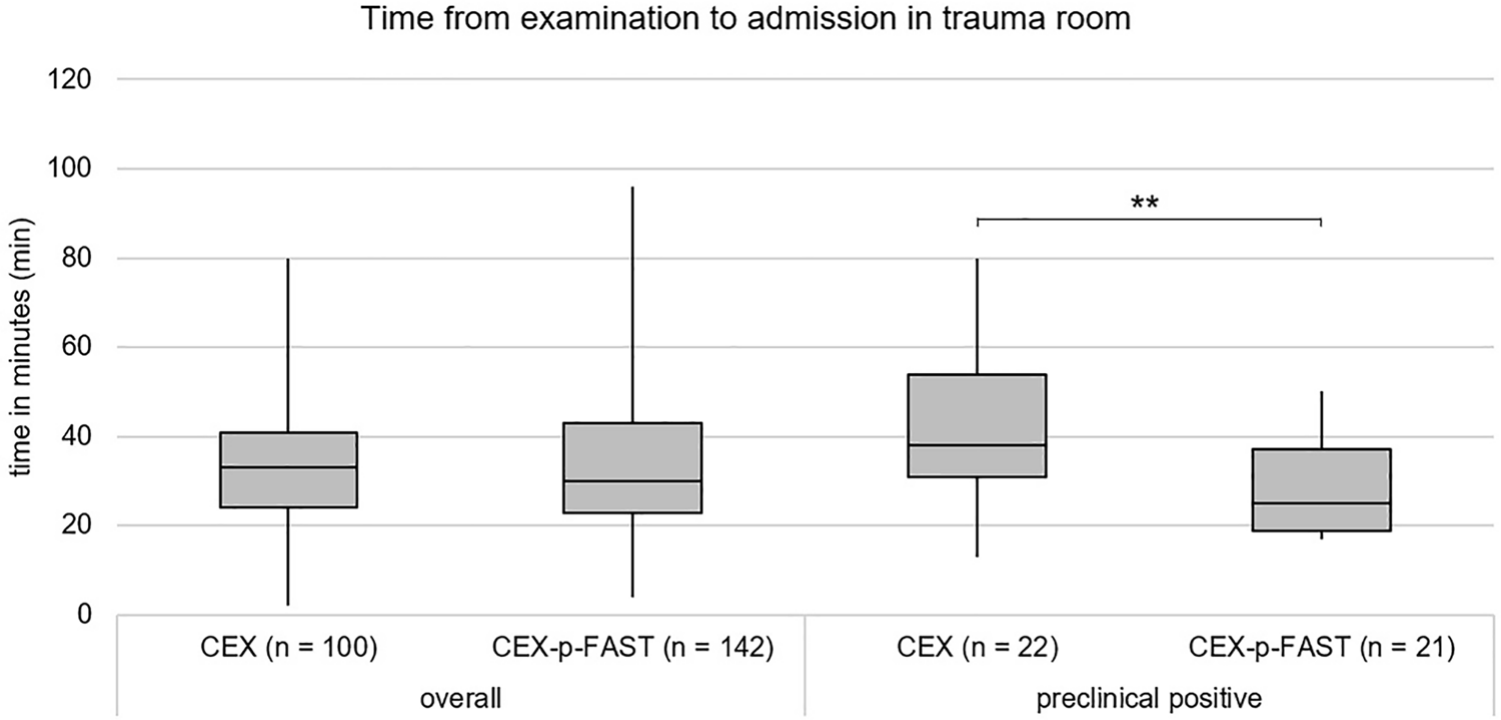
Time from examination to admission in trauma room. No significant difference in the time from the examination of the abdomen to admission to trauma room can be observed between the CEX (median 33 min; IQR 16 min) and CEX-p-FAST (median 30 min; IQR 20 min) groups. However, there is a significant difference between the two groups in the preclinical positive patient subset. In the p-FAST group (median 25 min; IQR 18 min), the time to admission to the trauma bay is significantly shorter than the CEX group (median 38 min; IQR 22 min; Mann–Whitney-*U*-Test *p* = 0.001; **p < 0.01)

Further, we evaluated the patients in the CEX with suspected positive findings or CEX-p-FAST groups with positive findings of free intra-abdominal fluid. In this subset, a significant decrease in the transfer time was observed in the CEX-p-FAST group in comparison with the CEX group (CEX 38 min vs. CEX-p-FAST 25 min; Mann–Whitney *U *test, *p* = 0.001; Fig. 2).

### Prehospital therapy and management

Patients in the CEX group in whom free fluid was suspected during the prehospital setting experienced changes in the therapy in 63.4%, in the admitting hospital in 68.2%, in communication with the admitting team in 77.3%, and in the management of transfer in 77.3%.

**Fig. 3:**
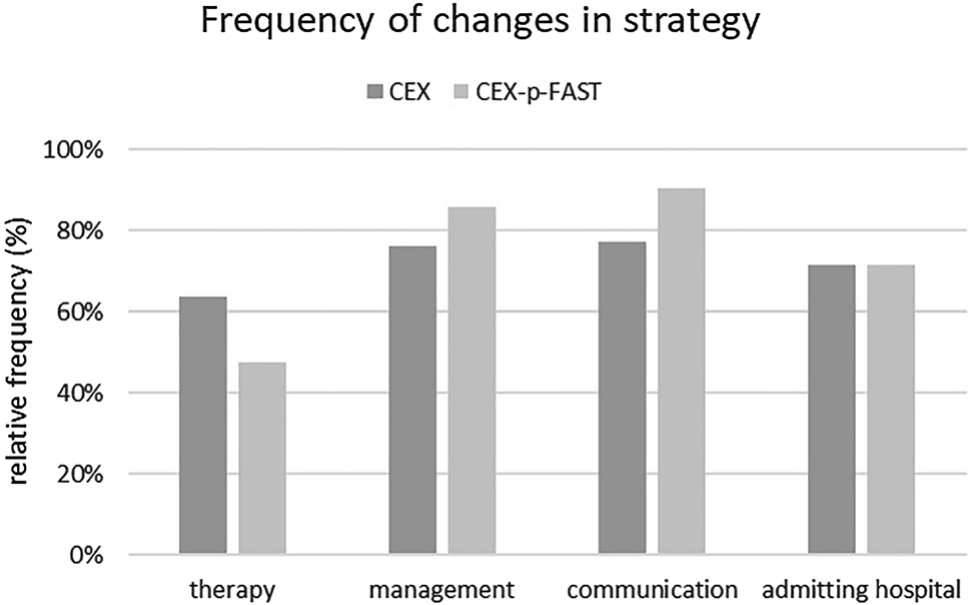
Frequency of changes in strategy. In preclinical cases suspected of abdominal injuries, changes in strategy, and treatment can be observed. In the CEX-p-FAST group, changes (i) in therapy at the scene in 47.6%, (ii) in admitting hospital in 71.4%, (iii) in communication with the admitting team in 90.48% and (vi) in management of transfer in 85.7% of the patients can be observed. In the CEX group, changes in therapy are seen in 63.4%, in admitting hospital in 68.2%, in communication with admitting team in 77.3%, and in management of transfer in 77.3% of the patients

In the CEX-p-FAST group, changes in the therapeutic strategy at the scene were observed in 47.6%, in the choice of the admitting hospital in 71.4%, in communication with the admitting team in 90.5% and in the management of transfer in 85.7% (Fig. 3).

### Time from prehospital exam to operative treatment

**Fig. 4:**
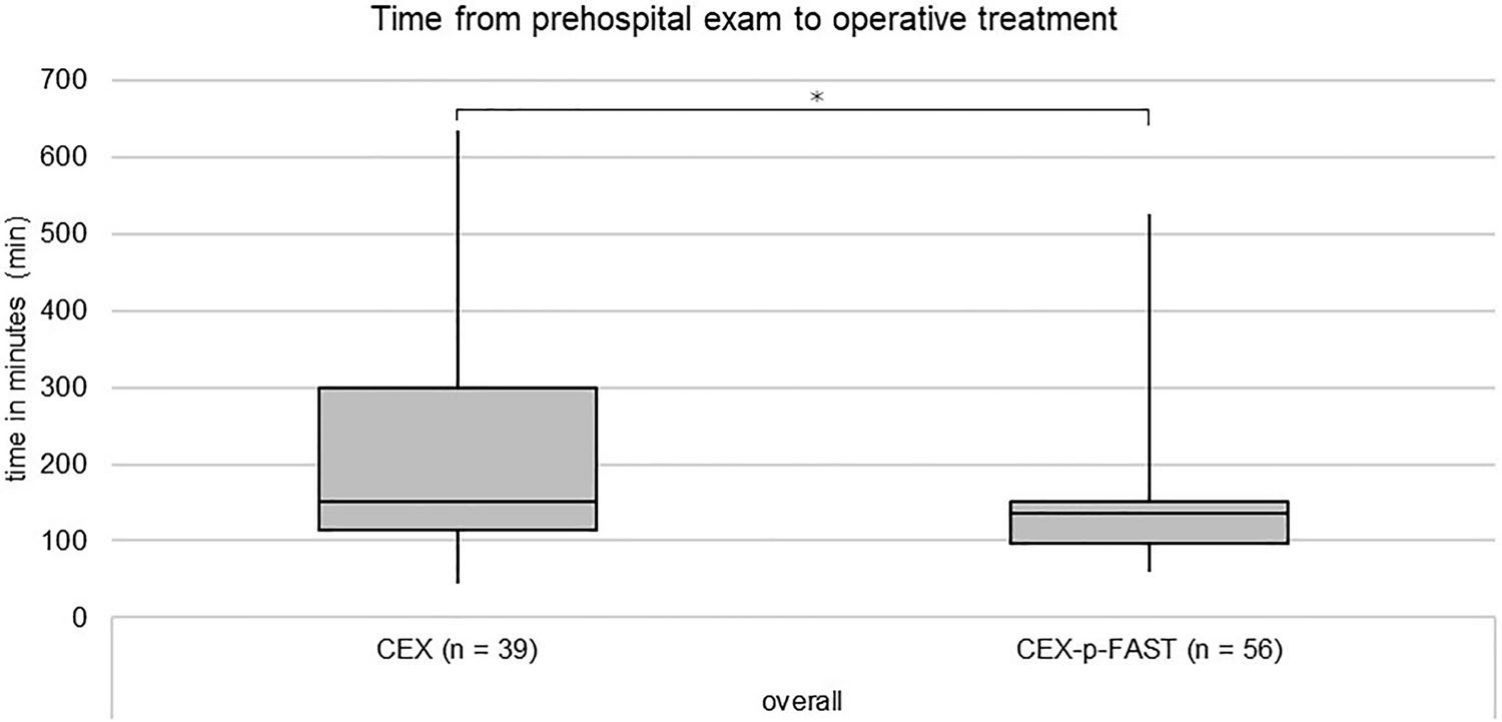
Time from prehospital exam to operative treatment. A significant decrease in time from prehospital examination to operative treatment can be observed in the CEX-p-FAST group (median 135 min; IQR 53 min) compared to the CEX group (median 150 min; IQR 185 min) (Mann–Whitney-*U*-Test *p* = 0.037)

A total of *n* = 105 patients needed an operation on the day of the trauma. Of all the patients who required surgery, *n* = 3 patients received emergent surgery in the trauma room, and *n* = 10 patients received emergent surgery after admission. Most of the operations were performed for the stabilization of the extremities (*n* = 46) or pelvic (*n* = 14) fractures. Laparotomy was performed in *n* = 7 cases and splenectomy in *n* = 6 cases. Regarding the laparotomies, in *n* = 1 case, a packing during laparotomy, in *n* = 1 case, an additional stabilization of a pelvic fracture and in *n* = 1 case, an additional stabilization of extremities and pelvic fractures were performed. In n = 95 patients, the time required to the operation was documented. Patients in the CEX-p-FAST group underwent a significantly shorter median time from the first prehospital examination to the beginning of the operative procedure (CEX 150 min vs. CEX-p-FAST 135 min; Kolmogorov–Smirnov *p* < 0.001 Mann–Whitney *U *test *p* = 0.037; Fig. 4).

## Discussion

In this study, we evaluated the effect of a prehospital FAST on the prehospital treatment, the disposition strategy, and the time course. We demonstrated that the test characteristics of the p-FAST plus CEX were better than those of CEX alone. Moreover, a shorter time interval to admission to the emergency department (ED) and the beginning of operative therapy was observed. However, the prehospital strategy changed in both groups when intraabdominal fluid was detected with p-FAST or suspected in CEX.

In hospital emergency medicine, ultrasound is an established diagnostic tool to assess injured patients quickly [14]. The use of ultrasound in prehospital settings has increased in recent years [15–17] due to improvements in image quality, size, weight, and the cost of portable ultrasound devices [18]. In our study, the acceptance of p-FAST increased during the study period. Initially, there were restraints regarding the use of p-FAST, with arguments like additional time at the scene. The rate of not performed p-FAST in the CEX-p-FAST group shortened constantly during the study period. However, we observed a cross-over rate of 30.8% during our interim analysis. In the CEX-p-FAST group, transport to the ED was significantly faster. Considering the high cross-over rate, confidence in the results of the ultrasound seems to be much higher than in those of the clinical examination. This caused a faster decision-making and shortened the time on scene. In consequence, the time to admission is significantly reduced in our study. During the CEX-p-FAST exam, the amount of free abdominal fluid was not estimated. Therefore, it could not be ruled out that the patient needs an urgent laparotomy. In this constellation, it could only be proven by a CT scan. Therefore, prehospital procedures were fastened. Thus, practically, despite the additional time for p-FAST, the overall prehospital time decreased substantially in the really critical patients and the time to admission in trauma room decreased significantly. Moreover, physicians increasingly used p-FAST against the decided study protocol due to patient safety reasons. Therefore, we decided to abort the study after the interim analysis due to ethical concerns, such as refusing p-FAST to patients with potential intraabdominal injuries and delaying time to operation, among others.

The sensitivity (94.7%) and specificity (97.6%) of the p-FAST in detection of free fluid in our setting are comparable to previous data [7, 18–20]. For FAST, a steep learning curve is already known. Walcher et al. reported that within 1 day of hands-on training, participants were able to perform ultrasound procedures at the scene of an accident with a high level of accuracy [15]. Shi et al. demonstrated that a short point of care ultrasound curriculum on emergency department physicians resulted in significant higher self-confidence and performance [21].

In addition to faster transport to the emergency hospital in the CEX-p-FAST group, we demonstrated a change in the prehospital management of cases in which free intraabdominal fluid was detected. Further, the transfer management, choice of admitting hospital, and communication with the admitting team were adjusted in the CEX-p-FAST group. This is similar to our previous results, as well as literature findings [7, 16]. Our previous data also showed changes in transfer management and choice of admitting hospital as well as therapy on scene [4]. In this previous study, most of the patients were transported by air rescue, and the changes were lower (e.g., if free intraabdominal fluid was detected with p-FAST, change in admitting hospital was observed in literature in 22% [4] vs. 71.4% in our study). Thus, the effect on change of patient treatment using ground-based emergency ambulances seems to be much higher.

Considering the high cross-over rate of 30.8% of p-FAST (*n* = 36) to CEX-p-FAST during the CEX-only weeks, the acceptance of ultrasound during prehospital settings increased rapidly. Previous studies have shown a change in the management of trauma patients due to the usage of prehospital ultrasound [4, 22]. An increasing acceptance of ultrasound has led to increased confidence in patient examination and the following adapted patient management. Thus, the German Society of Ultrasound in Medicine (DEGUM) has recommended the appropriate development of prehospital ultrasound in Germany [23]. Therefore, we recommend the p-FAST training for every EP as well as the usage of p-FAST in every patient with a blunt abdominal trauma. During conduction of the study, the extended FAST (E-FAST) was developed and widespread [24]. Regarding the sensitivity for thoracic injuries [24], we recommend further the use of the E-FAST protocol in preclinical scenario in place of the traditional FAST.

### Limitations

The high cross-over rate during the study period could have led to a selection bias in the patients. Moreover, the target recruitment was not reached due to the abort following the high cross-over rate. In this study, we showed a faster admission and shorter time to operative treatment. We discussed that this is caused by a higher confidence in ultrasound. Questioning the preclinical management and treatment, we only acquired the estimation of the EP if the result of the examination influenced the management or treatment. Detailed information about the preclinical therapy was not collected.

However, only level I trauma centers were chosen as admitting hospitals by the EPs. We showed a significant influence on choice of the admitting hospital. The data were assessed by a preclinical case report form filled by the emergency physician. In this form, it is explicitly asked if the sonography influenced the choice of admitting hospital as well as preclinical management and treatment. In this regard, it is asked among others if the patient was transported to the nearest hospital or to a level 1 trauma center. This option was also asked for the CEX-only group.

The data of this study were acquired between April 2007 and December 2009. Regarding many of the emergency ambulances were not equipped with ultrasound, the prediction and data were still currently and underline the recommendation of the DEGUM [23].

### Conclusion

A p-FAST, in combination with the CEX, showed high sensitivity and specificity for detection of free intraabdominal fluid, leading to optimized prehospital management, faster admission, and shorter time to operative treatment. p-FAST was increasingly accepted by the participating physicians during the study period leading to a cross-over rate of 30.8% since the physicians were concerned about patient safety.

## Data Availability

The datasets generated and/or analyzed during the current study are not publicly available due data privacy rules but are available from the corresponding author on reasonable request.

## Abbreviations

ATLS: Advanced Trauma Life Support
CEX: Clinical exam
CEX-p-FAST: Clinical exam and prehospital-focused assessment with sonography in trauma
CRF: Case report form
CT-abdomen: Computer tomography of the abdomen
DEGUM: German Society of Ultrasound in Medicine
ED: Emergency department
EMS: Emergency medical services
EP: Emergency physician
FAST: Focused assessment with sonography in trauma
ISS: Injury severity score
p-FAST: Prehospital-focused assessment with sonography in trauma
SD: Standard deviation
SPSS: Statistical package for the social science
